# Fatal miliary Coccidioidomycosis in a patient receiving infliximab therapy: a case report

**DOI:** 10.1186/1752-1947-1-79

**Published:** 2007-09-05

**Authors:** Mark P Rogan, Karl Thomas

**Affiliations:** 1 Department of Internal Medicine, Division of Pulmonary and Critical Care, University of Iowa Hospitals and Clinics, Iowa City, Iowa, USA

## Abstract

A 78-year-old white male from Iowa in the United States of America receiving the anti- tumor necrois factor (TNF) agent infliximab therapy for rheumatoid arthritis developed a cheek ulcer which failed to respond to empiric antibiotic therapy. He subsequently presented with progressive respiratory failure from miliary coccidioidomycosis which proved fatal. The patient vacationed in Arizona 6 months previously and likely contracted the organism there as Iowa is not an endemic area for coccidioidomycosis. Respiratory failure from miliary infiltration is an uncommon presentation of coccidioidomycosis. Physicians should be aware of the importance of travel history and potential for life-threatening coccidioidomycosis in patients receiving tumor necrosis factor inhibitors.

## Background

Tumour necrosis factor-α (TNF-α) is a cytokine that plays an important role in inflammation. In pathophysiological conditions, generation of TNF at high levels leads to the development of inflammatory responses that are hallmarks of many diseases. Anti- tumor necrosis factor agents are being increasingly used for immunomodulation in a wide variety of clinical conditions including inflammatory bowel disease, arthritides, psoriasis and atopic dermatitis. Early data suggests that they may have potential roles in vasculitides [1] and possibly sarcoidosis [2]. It is estimated that there are over 400,000 people currently on anti- TNF-α therapies worldwide [3]. These agents include: infliximab which is a chimeric mouse/human monoclonal IgG1 antibody directed at TNF; etanercept: which consists of 2 two copies of recombinant human TNF receptor p75 attached to the Fc portion of IgG1 and adalimumab-a fully human monoclonal antibody. Newer anti- TNF-α agents such as CDP571, CDP870 and onercept are currently being investigated in clinical trials [4]. Despite increasing popularity and broadening indications for usage, the anti-TNF agents have been associated with a wide variety of infections. We report a case of fatal miliary coccidioidomycosis in a patient receiving infliximab therapy.

## Case Report

**Figure 1:**
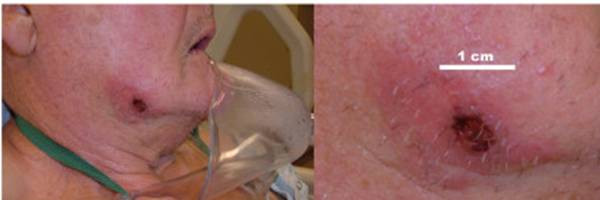
The ulcerated papule on the patient's right cheek with a close up view of the lesion.

A 78-year-old white gentleman from Iowa was diagnosed with sero-negative rheumatoid arthritis one year previously. He had been maintained on an immunosupressive regime consisting of methotrexate and the anti-tumor necrosis factor antibody, infliximab. A purified protein derivative skin test placed prior to initiation of infliximab was negative. His past medical history also included diabetes mellitus type II and hypertension. One-month prior to admission, he developed a slowly enlarging right cheek lesion (Figure 1). This was initially felt to be an area felt of localized cellulitis. He was treated with seven days of cephalexin and subsequently with amoxicillin/clavulanate without any significant response.

**Figure 2:**
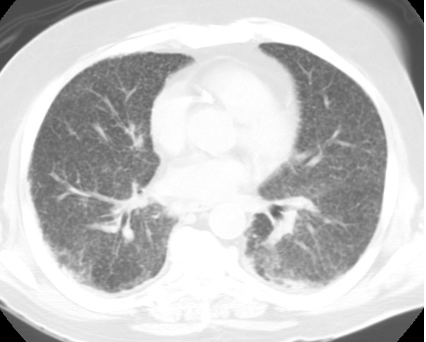
The patient's CT demonstrating bilateral, miliary interstitial infiltrate pattern.

He then presented to his local hospital with a 3-week history of progressive dyspnea on exertion, night sweats, fevers and 8 lbs weight loss. He had a cough productive of yellow mucoid sputum and was febrile to 38.5C. Investigations performed at the local hospital included a complete blood count that demonstrated a normochromic normocytic anemia with a hemoglobin of 10.2 gm/μl. His white blood cell count was elevated at 17,000 cells/L. Atypical pneumonia serology was normal. A trans-esophageal echo showed no vegetations. Blood and urine cultures were negative. His chest x-ray demonstrated diffuse bilateral infiltrates. A comuted tomography (CT) pulmonary angiogram study was negative for pulmonary embolus but demonstrated a diffuse, bilateral, miliary interstitial infiltrate pattern (Figure 2). He was treated empirically with levofloxacin and clindamycin for 7 days but his respiratory status continued to decline and he was transferred to the medical intensive care unit (MICU) at our University hospital for further management.

**Figure 3:**
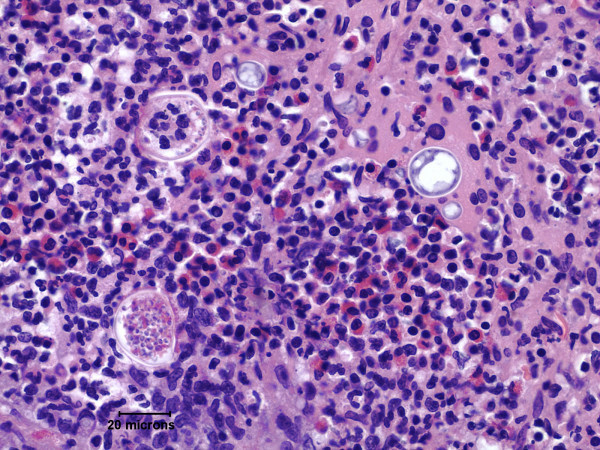
Hematoxylin and eosin staining of the skin biopsy (50x) demonstrating thick walled spherules containing endospores (arrows) consistent with Coccidiodes.

On arrival at the MICU, he was in respiratory distress with a respiratory rate of 32 breaths per minute. He was unable to complete sentences. His oxygen saturations were 91% on 80% oxygen by facemask and he was subsequently intubated and ventilated. He had a high-grade fever of 40.5 C. He underwent punch biopsy of the ulcerated papule on his right jaw-line. He also had bronchoscopy via the endotracheal tube. Bronchoscopy revealed normal appearing mucosa with widely patent airways and no significant secretions. Bronchoalveolar lavage (BAL) was performed times 2 with 20 mls per lavage with good return from the right middle lobe. Hematoxylin and eosin staining of both the cheek punch biopsy and the BAL revealed thick walled spherules containing endospores consistent with Coccidioides (figure 3). The patient was commenced on liposomal amphotericin (1.0 mg/kg per day) but despite this treatment, the patient ultimately died from respiratory failure 2 weeks later. On review of his travel history, the patient had lived in the mid-west all his life. However, 6 months prior to the onset of this illness, he spent 2 weeks visiting relatives in Phoenix, Arizona.

## Discussion

Coccidioidomycosis is a fungal disease caused by 2 nearly identical species, *Coccidioides immitis *and *C. posadasii*. It is endemic to areas of southwestern USA known as the Lower Sonora Life Zone. This semiarid zone encompasses southern Texas, Arizona, New Mexico, and much of central and southern California. Human infection occurs as a result of inhalation of arthroconidia. The majority of people exposed are asymptomatic but some develop an influenza-like illness and occasionally pneumonia. Dissemination is uncommon in immunocompetent hosts [5,6].

Coccidioidomycosis is associated with skin lesions such as the cheek lesion that our patient developed approximately 5-weeks prior to transfer to our ICU. We speculate that he contracted coccidioidomycosis in Arizona and that dissemination occurred over time whilst been treated with infliximab. Cutaneous involvement occurs by haematogenous dissemination to the skin or, much more rarely, from a primary cutaneous infection [7]. This lesion was mistakenly thought to represent a localized area of cellulitis and the patient was empirically treated with antibiotics for several weeks. Early biopsy and culture of this lesion may have led to a prompt diagnosis of coccidioidomycosis and perhaps the fatal outcome could have been prevented. This case highlights the consequences of a delay in diagnosis in patients receiving anti-TNF therapies.

Disseminated coccidioidomycosis has been previously described in immunosuppressed patients. For example, it is well recognized in the solid organ transplant population where it is thought to be a result of a primary infection or reactivation of latent infection [8]. Other groups of patients at risk for dissemination include pregnant women, African Americans and Filipinos, diabetics, HIV-positive patients and patients with lymphoma and other forms of immunosupression [7].

A recent meta-analysis of the anti-TNF antibody agents infiximab and adalumimab in 3493 patients with rheumatoid arthritis demonstrated increased risk of serious infections in 126 patients but only 1 case of coccidioidomycosis [9]. A multi-center study of rheumatologic patients from coccidioidomycosis endemic regions also demonstrated increased risk of coccidioidomycosis in patients treated with TNF antagonists [10]. In this study, 13 cases of coccidioidomycosis (12 associated with infliximab and 1 with etanercept) were identified among 918 patients being treated with TNF antagonist over a 5-year period. Interestingly, all patients presented with lobar pneumonia. None of the patients in this study had the bilateral, miliary infiltrates and progressive respiratory failure that we describe in this case. Only 5 patients were hospitalized: 2 died – one from meningeal coccidioidomycosis and one from central venous catheter related-sepsis.

In summary, anti TNF-α therapy is becoming more widespread in clinical use. These agents may predispose to disseminated, deep fungal infection and tuberculosis. Current recommendations prior to anti-TNF-α therapy include a chest x-ray, a tuberculin skin test and coccidioidomycosis serology. However serology may be an unreliable indicator of disease in such patients [5,10]. Coccidioidomycosis is one of the great imitators and may be the etiology of serous cavity infections such as pericarditis, empyema and peritonitis. It should be considered in the differential among patients presenting with atypical infections and a history of exposure to an endemic area. A careful travel history should be obtained in all patients prior to commencing anti TNF therapy. Physicians should maintain a high level of vigilance and promptly investigate any new signs or symptoms such as skin lesions in these patients. Antifungal prophylaxis with azole therapy is currently recommended for some high risk patients undergoing organ transplant from endemic areas [8]. Azole prophylaxis may have a role in patients on anti TNF-α therapy that live in endemic areas. In our case, the patient visited an endemic area for a brief period and then developed fatal coccidioidomycosis 6-months later. Further studies regarding the incidence of coccidioidomycosis in patients on anti TNF-α therapies and the effectiveness of azole prophylaxis in this group are needed before definitive recommendations can be made.

## Conclusion

We describe a case of fatal miliary coccidioidomycosis in a 78 year old white man taking the anti-TNF agent infliximab. The patient had vacationed briefly in an endemic area 6 months prior to presentation. He presented with a cheek lesion and rapidly progressive miliary lung infiltration and ultimately died from respiratory failure. This case highlights the importance of a travel history and the need for prompt investigation and diagnosis of cutaneous lesions in this patient population.

## Competing interests

The author(s) declare that they have no competing interests.

## Authors' contributions

MR is primarily responsible for drafting, literature search, submission and revision of the manuscript. KT is responsible for manuscript editing and advice on literature review. Both authors read and approved the final manuscript
